# Anti-libidinal Interventions and Human Rights

**DOI:** 10.1093/hrlr/ngab001

**Published:** 2021-02-10

**Authors:** Lisa Forsberg

**Keywords:** Anti-libidinal interventions, Articles 3, 8 and 12 of the European Convention on Human Rights, inhuman or degrading treatment, private and family life, right to found a family, consent

## Abstract

Anti-libidinal interventions (ALIs) are used in several jurisdictions to reduce male sex offenders’ libido. One common objection to these interventions holds that when offenders are either required to undergo them or offered to undergo them as an alternative to continued incarceration, ALIs violate recipients’ human rights. In this article, I examine this objection, which I call the human rights objection to ALIs, in relation to the European Convention on Human Rights (ECHR). Specifically, I examine the objection to ALIs in relation to Articles 3, 8 and 12 ECHR, which are the rights proponents of the human rights objection have identified as most relevant. I argue that the human rights objection in its current form fails to establish that ALIs violate recipients’ ECHR rights in respect of all these Articles.

Anti-libidinal interventions (ALIs)—sometimes colloquially referred to as ‘chemical castration’—are used in several jurisdictions in Europe and the United States to reduce male sex offenders’ libido.^1^ ALIs can be either hormonal drugs with testosterone-suppressing effects or non-hormonal drugs, such as anti-depressants or anti-psychotics, whose libido-reducing effects operate through other mechanisms.^2^ The mode of administration is generally either oral (as a drug regimen) or by injection.^3^ Legal arrangements for their provision vary considerably across jurisdictions:^4^ ALIs may be provided as part of treatment programmes offered to incarcerated offenders either on a voluntary basis or as a condition of parole,^5^ under mental health legislation (with or without recipient consent),^6^ or under dedicated ‘ALI statutes’ that explicitly provide for, and place conditions on, their use.^7^

ALIs remain controversial, however, especially when offenders are either compelled to undergo the intervention or offered it as an alternative to (continued) incarceration. One common objection holds that, when used in either of these ways, ALIs violate recipients’ human rights. Call this the human rights objection to ALIs (HRO). Although common, the HRO is rarely explicitly or precisely stated. This makes the nature of the objection unclear. It is hard to know, for example, whether the human rights allegedly violated by ALIs are *moral* or *legal*, whether all or just some ALIs are incompatible with human rights and what features of ALIs or the way in which they are provided cause them to violate recipients’ human rights, when they do so.

Clarifying the HRO is important for at least two reasons. First, ALIs are already used in many jurisdictions, under a variety of legal arrangements, and other jurisdictions are considering their use.^8^ Second, we may have reason to expect biomedical interventions that target aggression, impulsiveness and other risk factors for criminal conduct to become more common, as we learn more about how such dispositions may be controlled^9^ and given the limitations and costliness of traditional ways of dealing with offenders. The extent to which such interventions are compatible with human rights seems a *prima facie* important factor to consider when determining the conditions under which the state could permissibly offer or require offenders to undergo such interventions.^10^ Getting clear on what is at stake in the HRO may help us to determine the permissibility of biomedical interventions used for crime-prevention purposes more generally.

This article aims to clarify when, if ever, non-consensual ALIs provided to sex offenders may violate recipients’ human rights, and, if they do, the features of ALI provision by virtue of which they do so, with reference to the legal human rights enshrined in the European Convention on Human Rights (ECHR). I focus on *non-consensual* ALIs because proponents of the HRO generally seem to take the absence of valid recipient consent to be a human rights-violating feature of ALIs,^11^ and because non-consensual ALIs seem the hardest to defend. If non-consensual ALIs can be defended from the HRO, then, consensual ALIs seem to stand a good chance of being defended from it, too. But my purpose here is not to defend ALIs, or particular uses of it. Rather, it is to clarify the circumstances in which non-consensual ALIs are incompatible with the ECHR.

The focus on the ECHR may require further explanation and justification. Philosophical accounts of human rights have tended, when engaging with international human rights law or the practice of human rights, to focus on the Universal Declaration of Human Rights (UDHR).^12^ But we have reason to focus on the ECHR instead. First, the UDHR is not legally binding. However, the rights in the UDHR are closely mirrored in the ECHR, which is legally binding on the Contracting Parties. Certainly, this is true of all the rights in the ECHR that we will engage with here.^13^ Considering the ECHR, then, involves engaging with rights that are *prima facie* similar to those enshrined in the UDHR, but in a form that has legally binding character.^14^ Second, a considerable body of case law further specifies the ECHR rights that bear on the provision of medical interventions such as ALIs.^15^ Third, some of the most well-developed (legal) accounts of the HRO invoke the ECHR, arguing that (at least non-consensual) ALIs may breach it.^16^ Therefore, versions of the HRO based on the ECHR seem to represent the most well-developed version of the objection. The legal rights protected by the ECHR articles invoked by HRO proponents also mirror legal rights that have been taken as important (and potentially threatened by biomedical interventions used for crime-prevention) in other jurisdictions (such as the United States) and moral rights.^17^ The HRO and responses to it therefore seem to be generalisable beyond the ECHR.

I begin by attempting to specify the HRO to ALIs (section 1). I then proceed to examine how the HRO fares with reference to the ECHR, examining in turn Article 3 (section 2), Article 8 (section 3) and Article 12 (section 4)—the rights proponents of the HRO typically identify as most relevant to the permissibility of ALIs.^18^ I argue that the human rights objection in its current form fails to establish that ALIs violate recipients’ ECHR rights in respect of all these articles.

## 1. THE HUMAN RIGHTS OBJECTION TO ALIs

The HRO may seem, to many, intuitively plausible. The history of psychiatry gives us terrifying examples of non-consensual ‘treatments’ imposed on individuals detained primarily for ‘socially deviant’ behaviour, and ‘chemical castration’ has been used in morally reprehensible ways in the past.^19^ It is unsurprising, then, that arguments to the effect that ALIs violate human rights have been put forward in academic,^20^ policy^21^ and popular^22^ fora. Although the HRO is rarely properly specified, we get a flavour of what is at issue from some examples of human rights being invoked in objections to ALIs.

Amnesty International stated in reference to the enactment of a law providing for non-consensual ALI provision in Indonesia in 2016 that:

Imposing [ALIs] by law without informed consent as a punitive measure would be a cruel, inhuman and degrading punishment. Forced chemical castration is a violation of the prohibition on torture and other cruel, inhuman or degrading treatment or punishment under international law.^23^

Amnesty International Moldova stated in 2012 that legal amendments providing for ALI use:

undermined the basic right to physical and mental integrity, i.e. the right not to be tortured or ill-treated in any form. International human rights treaties expressly ban torture and inhuman or degrading treatment .... It is clear that imposition of forced medical procedures amounts to a recourse to inhuman treatment ... forced chemical castration ... is incompatible with human rights, which are the foundation of any civilized democratic society’.^24^

The European Committee for the Prevention of Torture, in its review of practices in EU member states and in particular the Czech Republic’s administration of medical interventions, including surgical castration and ALIs, to incarcerated individuals, stressed that:

... medical interventions, and in particular medical interventions which have irreversible effects on persons deprived of their liberty, should as a rule only be carried out with [recipients’] free and informed consent. Given the particularly vulnerable position of persons deprived of their liberty in this regard, it should be ensured that the patient’s consent is not directly or indirectly given under duress and that the patient receives all the necessary information when making his decision. Furthermore, the Committee considers that the concept of ‘free and informed’ consent is hardly reconcilable with a situation in which the options open to an individual are extremely limited: surgical castration or possible indefinite confinement in a psychiatric hospital. ^25^

As for academic commentators, Bernadette Rainey argues that:

If consent is free and informed then chemical castration may be rights-compliant, but if this is not the case or the state decides to have a compulsory regime on competent (or incompetent) persons then issues may arise under the ECHR.^26^

Karen Harrison and Rainey argue elsewhere that ‘[i]f [ALI] treatment is given with free and informed consent, then it is unlikely to be found to be in violation of human rights legislation’.^27^

The assumption that recipient consent is necessary for human rights-compliant administration of ALIs is perhaps unsurprising. The ECHR treats consent as central in respect of medical interventions more generally,^28^ and consent is central in English law’s regulation of medical interventions.^29^ Moreover, both sides in the debate over the *moral* permissibility of biomedical crime-preventing interventions such as ALIs have tended to accept (at least implicitly) a consent requirement in respect of such interventions, also when they are used for crime-prevention (rather than therapeutic) purposes.^30^

Some HRO proponents go further, objecting not just to non-consensual ALIs, but also to ALIs offered as an alternative to incarceration or condition of parole, arguing that:

If the offender is given the choice between a course of testosterone-reducing medication and a period of incarceration, it is likely that the offender will be coerced into choosing the treatment, on the basis that it is the lesser of two evils. Coerced consent may also occur if the offender is led to believe that participation in a pharmaceutical programme will enhance his chances of parole.^31^

I shall not engage with the validity of consents of this kind here. I will merely note that *if* such ‘consents’ are indeed invalid, my arguments here will apply to them, too.

In the sections that follow, I will argue, *pace* those who have defended the HRO, that non-consensual ALIs are likely often to be compliant with the ECHR and that when they are not, it will not generally be for want of offender consent. The HRO as it stands is likely to fail and those who wish to object to the use of ALIs would do better to find a different objection that does not rely on consent.

## 2. ALIs AND ARTICLE 3 ECHR

Let us first consider a version of the HRO according to which non-consensual ALIs violate recipients’ human rights under Article 3 ECHR. Article 3 holds that ‘[n]o one shall be subjected to torture or to inhuman or degrading treatment or punishment’ and imposes an obligation on member states to protect individuals from such treatment. Unlike many other Convention rights, Article 3 is absolute:^32^ ‘the Convention prohibits in absolute terms torture or inhuman or degrading treatment or punishment, irrespective of the victim’s conduct’,^33^ and ‘it is of the essence of the state’s obligation not to subject any person to suffering which contravenes Article 3 that the ends cannot justify the means’.^34^ That Article 3 is absolute means that when the article is engaged, nothing—no circumstances, conduct of the victim or grounds, such as public protection—will *justify* its violation.^35^

Interference must reach a minimum level of severity to engage Article 3. Whether this minimum severity threshold has been reached is determined by taking into account characteristics of the intervention (its nature, duration, effects and so forth) and of the victim (their health, sex, age,^36^ decision-making capacity,^37^ etc.). In addition to the nature of the intervention itself, the *context* in which it takes place is relevant to establishing Article 3 breach.^38^

We may think it unlikely that ALIs would reach Article 3’s severity threshold. First, their anti-libidinal effects are typically non-permanent and reversible.^39^ Second, in contrast with surgical castration, which may exceed the severity threshold, ^40^ the mode of administration is often relatively non-invasive; ALIs are usually administered as drug regimens or by repeated injection.^41^ Third, the European Court of Human Rights (ECtHR) noted in *Jalloh v Germany* that the Convention does not prohibit forced medical intervention per se,^42^ and it is accepted that both treatment and punishment involving *some* burdens can be imposed on individuals by the state without breaching Article 3.^43^

HRO proponents may point out that at least some ALIs interfere with bodily integrity (often repeatedly), and note that Article 3 breach has previously been found also in respect of seemingly non-severe bodily interference, such as a slap in the face of a detainee, due to the context in which the interference takes place (detention).^44^ They may also observe that while the main or intended effects of ALIs are non-permanent and reversible, some of their side-effects may not be. If ALIs affect recipients’ health negatively,^45^ or its administration or side-effects cause feelings of anguish, humiliation, fear or inferiority,^46^ the ECtHR might be more likely to find an Article 3 breach. ALIs carry numerous side-effects, some of which are quite serious, including osteoporosis, liver damage, metabolic abnormalities, thrombophlebitis, cardiovascular disease, depression, fatigue, hypersomnia, lethargy and weight gain.^47^ Possible side-effects also include ‘feminising’ effects, such as breast growth and lactation,^48^ which, some have argued, may be degrading.^49^ Moreover, ALIs suppress general sexual functioning and not just criminal sexual conduct and may also affect recipients’ personality, inducing symptoms such as fatigue, depressive symptoms or disorders, emotional disturbances, anxiety and aggression reduction.^50^ Given this, HRO proponents may argue, at least *some* ALIs, in some circumstances, may reach the threshold for Article 3 engagement. It may be responded that the ECtHR has previously rejected claims to the effect that an intervention’s serious negative side-effects met the severity threshold for Article 3 engagement (in the case of non-consensual neuroleptic drugs).^51^ This may suggest that side-effects of ALIs, too, would be unlikely to engage Article 3, unless they were very serious or long term.^52^ Moreover, any ‘feminising’ physical effects may be corrected through surgery.

But HRO proponents may again point to the context in which ALIs are likely to be administered (under detention, without consent and so on) and argue that this makes breach more likely. They may also argue that the *length* of the intervention (including side-effects and effects on general sexual functioning) sets ALIs apart from other interventions accepted as ECHR compliant. ALIs do not ‘cure’ offenders and may therefore need to continue for long time periods.^53^ If it is the permanence (or long lastingness) of the effects (on recipients’ sexual function and so on) that causes surgical castration to violate Article 3, then long-term pharmaceutical ALIs may also reach the severity threshold, if they, too, have these effects. But this restricts the scope of the HRO in respect of Article 3 engagement to a subset of ALIs: very long-term or time-unlimited ones with severe effects/side-effects.

Moreover, crucially, ALIs may fall outside the scope of Article 3 if they are accepted to be a ‘medical necessity’. The leading case here is *Herczegfalvy v Austria,* which held that the applicant’s being administered sedatives and forced fed against his will while detained in psyschiatric hospital did not violate his Article 3 rights since ‘the evidence before the court [was] not sufficient to disprove the government’s argument that, according to the psychiatric principles generally accepted at the time, medical necessity justified the treatment in issue’.^54^ The Court held that ‘as a general rule, a measure which is a medical necessity cannot be regarded as inhuman or degrading’, but noted that ‘[t]he court must nevertheless satisfy itself that the medical necessity has been convincingly shown to exist’.^55^ Might ALIs qualify as medically necessary? If ALIs were provided to all offenders who had committed a particular criminal offence, without regard for diagnosis (if any) and suitability to receive ALIs, HRO proponents might have a solid case for Article 3 breach (assuming the severity threshold had been reached).^56^

Putting blanket provision of this kind to one side, however, it seems that ALIs may qualify as medically necessary.^57^ English courts have accepted sex offending as a manifestation of mental disorder^58^ and ALIs as treatment for such disorder.^59^ Moreover, the Mental Health Act 2007 amended the Mental Health Act 1983 such that the test is merely *appropriate treatment being available*, rather than treatment being available that ‘is likely to alleviate or prevent deterioration in the patient’s condition’, as was the requirement prior to the amendments.^60^ The ECtHR held in *Dvořáček v Czech Republic* that sexological treatment (including ALIs) administered during the applicant’s detention in a psychiatric hospital did not amount to inhuman and degrading treatment in violation of Article 3, since although the measures undoubtedly caused the applicant discomfort, they were justified by his state of health and his conduct.^61^ HRO proponents may object that, given the paucity of evidence of their effectiveness, ALIs cannot plausibly qualify as medically necessary.^62^ Interventions have previously failed the *Herczegfalvy* test for lack of robust evidence of effectiveness. For example, an anti-psychotic medication failed the test on grounds that its benefits were ‘decidedly speculative’.^63^ The same could perhaps be said of ALIs. But the ‘medical necessity’ standard may be less demanding than it first appears. In *R (on the application of B) v Haddock (Responsible Medical Officer)*, which considered the medical necessity standard under Article 3, an intervention was found ‘convincingly shown’ to have been a medical necessity when it was believed by clinicians to be more likely than not to succeed.^64^ Moreover, the fact that a responsible body of medical opinion exists, which disputes that a particular intervention is a medical necessity or in patients’ best interests does not in and of itself cause the intervention to fail the test.^65^ The belief among some clinicians that ALIs are medically necessary (in respect of some offenders), then, might be sufficient for them to be accepted as such, even if clinical opinion is divided and the evidence inconclusive.^66^ My claim here, then, is not that ALIs are medically necessary according to some strict standard, but merely that given that the relevant standard is not very demanding, some ALIs may meet it.^67^

HRO proponents may counter that ALIs ought not be construed as medically necessary, not just because their effectiveness is limited, but also because their purpose is more appropriately understood as correctional than medical or therapeutic. Some of the quotes in section one above indicate that some HRO proponents conceive of ALIs as correctional rather than medical/therapeutic interventions.^68^ We may agree with HRO proponents that ALIs are, at least sometimes, correctional, or a mixture of medical and correctional, interventions. In England and Wales, offenders may receive ALIs (with or without consent) after having been involuntarily committed and treated under mental health legislation as part of criminal sentences, their detention justified on grounds of it being ‘necessary ... for the protection of other persons that [they] should receive such treatment’, and the Secretary of State for Justice may restrict clinicians’ discretion in respect of decisions regarding leave or release. ^69^ These features of ALI provision seem to suggest a correctional objective. But if ALIs are correctional interventions, it seems less plausible that their provision is *medically necessary*.^70^

Whether the purpose of ALIs is medical or correctional is relevant to determining what conduct gave rise to the alleged violation under Article 3: ‘inhuman or degrading *treatment* or *punishment*’. In *Dvořáček,* the ECtHR emphasises this distinction, considering that, ‘the protective sexological treatment imposed on the applicant had been intended to protect him and therefore *had not constituted a “punishment”* within the meaning of article 3 .... The question examined by the Court was therefore whether the conditions to which he had been subjected had in themselves amounted to “inhuman” or “degrading” *treatment’*.^71^ ALIs may be more likely to be in breach of Article 3 if administered for punitive or correctional (rather than medical/therapeutic) purposes and in the absence of medical supervision, without recipient consent, and if they are administered in a way that humiliates or debases the offender or disproportionate force or restraint is used.^72^ It may be that the Court first seeks to establish whether the purpose or character of the intervention is medical or correctional, since this is a relevant factor for the ECtHR in analysing the context in which the alleged violation takes place,^73^ and second, it examines whether, in case of interventions with a therapeutic purpose or character, they are in fact medically necessary (employing the *Herczegalvy* test). If an intervention is accepted as both therapeutic in character or purpose and medically necessary, the ‘general rule’ established in *Herczegalvy* applies such that the intervention being regarded as ‘inhuman or degrading’ is less likely. If the intervention is considered to have a correctional, rather than therapeutic, character or purpose, however, this general rule does not apply and the medical necessity test cannot justify its provision, since Article 3 does not allow for any type of proportionality or necessity analysis that weighs up how necessary a (degrading/inhuman) punishment is to achieve a public aim such as crime-prevention.

If this is the case, however, it seems that HRO proponents may be on stronger ground not arguing for a consent requirement in relation to Article 3, instead maintaining that ALIs should be conceived of as correctional rather than therapeutic interventions and their provision therefore not considered medically necessary. Insisting on a consent requirement seems to involve conceding that ALIs are therapeutic rather than correctional interventions, since it is in respect of the former, and not the latter, that a consent requirement is generally accepted.^74^ It may seem, then, that at least in respect of Article 3, HRO proponents would be better off abandoning their focus on consent. Or that, if they wish to retain their focus on consent, HRO proponents are better off focusing on another Article of the ECHR.

## 3. ALIs AND ARTICLE 8 ECHR

Let us now turn to a version of the HRO according to which non-consensual ALIs violate recipients’ human rights under Article 8 ECHR. Article 8(1) holds that ‘[e]veryone has the right to respect for his private and family life, his home and his correspondence’, and Article 8(2) holds that ‘[t] here shall be no interference by a public authority with the exercise of this right except such as is in accordance with the law and is necessary in a democratic society in the interests of national security, public safety or the economic wellbeing of the country, for the prevention of disorder or crime, for the protection of health or morals, or for the protection of the rights and freedoms of others’.^75^A consent requirement seems more plausible in respect of Article 8, since it is accepted as the Convention right that most explicitly and robustly protects personal autonomy,^76^ and bodily integrity is generally considered an essential part of or precondition for personal autonomy.^77^ In *Pretty v United Kingdom*, the ECtHR reiterated that ‘the concept of ‘private life’ is a broad term not susceptible to exhaustive definition’ but that ‘[i]t covers the physical and psychological integrity of a person’.’^78^ This is in turn generally taken to generate a requirement to seek competent individuals’ valid consent before administering interventions interfering with their bodily integrity.^79^ ALIs administered to offenders without their consent are likely, therefore, to engage Article 8. In *X v Austria,* the ECtHR held that ‘[c] ompulsory medical intervention, even if it is of minor importance, must be considered an interference with [Article 8 ECHR]’.^80^ In *YF v Turkey*, a gynaecological examination of the applicant while in police custody was held a violation of her Article 8 rights, since ‘any such interference with the physical integrity of a person had to be prescribed by law and required the consent of that person’.^81^ The assisted death cases that have come before the ECtHR and set out a right to decide ‘how and when to die’ could also plausibly be generalised as a right to take decisions that concern one’s bodily autonomy more generally.^82^

It might be argued that while administering ALIs to offenders without their consent seems a *pro tanto* interference with their autonomy, doing so does not obviously diminish their autonomy all things considered. For reducing an offender’s sex drive may render him more capable to resist his urges and better able control his thoughts and therefore able to think and act more freely. If ALIs have this effect, they may be *autonomy-promoting*.^83^ HRO proponents may remain unconvinced by this argument, however, or they might think that bodily integrity is so fundamental to autonomy and to offenders’ Article 8 rights that interference with it is impermissible even if it promotes recipients’ autonomy all things considered.^84^ We have a right to be free from non-consensual interference with our bodies in other situations such as standard medical treatment, they might say, even when such interference would benefit us and increase our autonomy.^85^

We might respond that this presupposes more robust protection for autonomy/bodily integrity than is supported by Article 8 ECHR. Crucially, Article 8(1), even when engaged, offers only a qualified right to personal autonomy. Interferences with an individual’s Article 8 rights may be *justified* and thus compliant if they: (i) pursue a legitimate aim, are (ii) in accordance with the law and (iii) necessary in a democratic society. Condition (iii) turns on proportionality: ALIs can be necessary in a democratic society only if they are proportionate to the end they aim to achieve. I grant (ii) for the sake of argument.

In respect of (i), ALIs may serve several legitimate aims. They may be accepted as serving the aim of protecting the recipient’s own health, especially if he suffers from a treatable mental disorder,^86^ since as noted above, English courts accept that sex offending may be a manifestation of mental disorder^87^ and ALIs treatment for such disorder.^88^ In general, however, the individual’s right to refuse interventions interfering with her bodily integrity enjoys strong protection.^89^ ALIs may therefore be more readily accepted as compliant when furthering other legitimate aims. The ECtHR has previously accepted non-consensual medical interventions such as blood tests, vaccinations and screening programmes as justified on grounds of protection of the rights and freedoms of others,^90^ prevention of disorder or crime,^91^ public safety^92^ and the preservation of public order.^93^ ALIs employ similar means (injections, drugs) and they could arguably be said to pursue any of these legitimate aims, since ‘[s]exual offending is a serious social problem, a public health issue, and a major challenge for social policy’.^94^ But we may be on even stronger ground arguing that ALIs pursue the aim of the prevention of disorder or crime, since sex offences are serious crimes generally warranting severe responses.

In respect of condition (iii), focusing on proportionality, ALIs may, even if administered in pursuit of a legitimate aim, constitute a disproportionate interference with recipients’ rights. The proportionality test employed by the ECtHR to determine whether a (qualified) right has been violated has tended to be implicit,^95^ but the ECtHR has held that a proportionality requirement inheres in the Convention as a whole, also when not explicit.^96^ Moreover, the analysis employed by the ECtHR corresponds to that used by English courts, who have been more forthcoming.^97^ For example, the proportionality inquiry was explicitly stated in *R (Aguilar Quila and another) v Secretary of State for the Home Department:*^98^

(a) is the legislative objective sufficiently important to justify limiting a fundamental right? (b) are the measures which have been designed to meet it rationally connected to it? (c) are they no more than are necessary to accomplish it? (d) do they strike a fair balance between the rights of the individual and the interests of the community?^99^

Similarly, in *Bank Mellat*, Lord Reed broke down the test in four steps:

(1) whether the objective of the measure is sufficiently important to justify the limitation of a protected right, (2) whether the measure is rationally connected to the objective, (3) whether a less intrusive measure could have been used without unacceptably compromising the achievement of the objective and (4) whether, balancing the severity of the measure’s effects on the rights of the persons to whom it applies against the importance of the objective, to the extent that the measure will contribute to its achievement, the former outweighs the latter.^100^

The proportionality requirement places constraints on the arrangements under which ALIs can be permissibly provided. It would, for example, rule out the blanket provision of ALIs in response to certain crimes, regardless of offenders’ suitability or medical needs. This is because blanket provision would allow for ALIs being provided to offenders (interfering, we assume, with their Article 8 rights) for whom they could not reasonably be expected to have any of the intended effects. There would therefore not be any rational connection between the measure (ALIs) and its (legitimate) aim (reduced risk of sex offending).^101^

Blanket provision aside, however, it does not seem obvious that ALIs would be a disproportionate measure. We want, presumably, to allow for some kinds of responses to criminal offending that go beyond how we would treat non-offenders. Sex offences are serious crimes that will generally warrant incarceration. The fact that potential ALI recipients will often, due to having been convicted of such crimes, already be liable to be treated in certain ways in which non-offenders could not legitimately be treated affects the balancing of the interests of the individual and the public under Article 8(2).^102^ It is thus not sufficient to demonstrate that we *in general* have a right against non-consensual interference with our bodily integrity to have a compelling argument against ALI use in sex offenders. We may accept (as most of us do) that we generally have a right to bodily integrity, but note that we also generally have a right against the restrictions of freedom of movement and association involved in incarceration.^103^ Incarceration of criminal offenders is not justified on grounds that we do not generally have a right to freedom of movement and association, but rather on grounds that the offender’s conduct makes him liable to be treated in certain ways in which it would not be permissible to treat him, but for that conduct.^104^

The justification for treating offenders and non-offenders differently is (generally) taken to be along the lines that the offender’s criminal conduct causes some of his rights to lose some of their protective force, for example, because he waives some of his rights by committing a criminal offence, or because his offence activates an exception clause already built into those rights or confers on other individuals certain rights to restrict some of the offender’s rights (which must then be balanced against the offender’s persisting rights against having his rights restricted).^105^ The structure of Article 8 seems to reflect one or more of these ideas. It sees interferences with an offender’s rights as justified when pursuing a sufficiently important public interest, such as crime-prevention or the protection of others and when the interference is proportionate to the importance of the public interest pursued and is taking place in response to a certain act by the offender. The question, then, is *which* of the offender’s rights are affected in this way by his criminal conduct, and *why these* and not other rights can be permissibly restricted in response to it. *Prima facie*, it seems that the kinds of considerations that justify restrictions on our freedom of movement could also justify restrictions on our freedom from interference with our bodily integrity.^106^

Proponents of the HRO may argue that the right to bodily integrity is *more robust* than other rights, such that it is not susceptible to being affected by our criminal offending in the way or to the degree our right to freedom of movement is affected by criminal offending. They may point out that while we generally accept that criminal offending makes it permissible for the state to impose *some* measures it could not permissibly have imposed but for that offending, few would hold that criminal offending renders us liable to *all* or *any* forms of restrictions of our rights. An individual’s criminal offending, even when serious, is not typically thought to render it legitimate for the state to torture him, for example. This might be because some of our rights, such as our rights not to be tortured, are *more robust* than some of our other rights, such as our right to freedom of movement.^107^ Article 3 seems to represent a right of this kind, for under it we have a right to be free from torture and inhuman or degrading treatment *irrespective* of whether a good justification for overriding exists and irrespective of our conduct.^108^ HRO proponents may argue that our right to bodily integrity is like the right not to be tortured or like Article 3. But, as we saw in section 2, Article 3 does not protect bodily integrity per se*,* it protects against invasions of it of a particular severity. And the Article that comes closest to protecting bodily integrity per se—Article 8—offers only qualified protection and accepts that interferences can be justified on either of several grounds. The ECHR framework does not, then, appear to regard the right to bodily integrity as akin to the right not to be tortured.

It may be responded that even if the right to bodily integrity is not quite like the right not to be tortured, it is nevertheless more robust than, for example, the right to freedom of movement. Therefore, ‘it takes a greater deviation from normal circumstances for the right to bodily integrity to lose its protective force than for the rights to freedom of movement and association to lose their protective force; it takes more to render oneself liable to impositions on bodily integrity than to render oneself liable to impositions on free movement and association’.^109^ But it does not seem obvious that this is the case. To make this move, HRO proponents would need to provide an additional argument for bodily integrity having a special status or our interest in it being much more important than our interest in freedom of movement.

In the absence of such an argument, HRO proponents might try to hang their objection to interventions interfering with bodily integrity on some other consideration. For example, they might rely on an argument from harmfulness instead. They may argue that even if the *mode of administration* of ALIs is relevantly similar to those the ECtHR has previously accepted as justified (blood tests, etc.), either the intended effects (loss of sexual desire, etc.) or side-effects (liver damage, osteoporosis, etc.) of ALIs set them apart. The harmfulness of these effects may suffice for ALIs to fail the proportionality test. HRO proponents may also argue that potential ‘feminising’ side-effects, such as breast growth and lactation, might be degrading and therefore disproportionately harmful.^110^ But it does not seem obvious that non-consensual interferences with someone’s bodily integrity (of the kind involved in ALI use) typically entail more serious harm than restrictions of freedom of movement and association (of the kind involved in incarceration). For restrictions of freedom of movement and association involved in incarceration have harmful effects too (albeit some of them of a different kind): restricting offenders’ ability to form and maintain personal relationships including sexual ones, hindering the pursuit of most life-plans and most sources of well-being and typically causing significant distress.^111^ The same seems to be true of other values on which objections to interferences with bodily integrity might be hanged. Whether it is argued that interferences with one’s bodily integrity will be *experienced* as more harmful^112^ or would be a greater threat to one’s agency or sense of agency^113^ or sense of identity and self-control,^114^ it is not obvious that incarceration would not affect offenders in similar ways. The fact that the ALI recipients we are concerned with here are already liable to being subjected to some burdensome responses to their offending seems to generate at least *some* obligation for HRO proponents to engage with the respective burdensomeness of ALIs and traditional responses, if they are to establish that ALIs are a disproportionate response to criminal offending.^115^

There is, however, another reply available to HRO proponents. They might again point to the limited evidence of ALI effectiveness and argue that if ALIs are ineffective in achieving the desired (legitimate) end, while imposing potentially quite severe side-effects on recipients, this may render them a disproportionate response to sex offending.^116^ Indeed, the evidence of ALIs’ effectiveness may be so limited that HRO proponents could argue that they would fail the second step of the proportionality inquiry—the rational connection limb, that is, whether the relevant measure is adequate to achieve the pursued aim—without even needing to consider whether ALIs are proportionate *strictu sensu* (step four of the proportionality inquiry). But this reduces the HRO to an empirical objection, which holds only as long as the evidence of ALI effectiveness remains sparse.^117^ Moreover, if we take the desired (and, suppose, legitimate) aim of both ALIs and traditional responses such as incarceration and behavioural sex offender programmes to be reducing the risk of recidivism (post-release), we may grant that the effectiveness of ALIs in achieving this end is limited, but point out that the alternatives are not particularly effective, either.^118^ If incarceration is a proportionate response, despite its serious problems, it seems unlikely that *no* ALIs could survive the proportionality analysis.^119^

## 4. ALIs AND ARTICLE 12 ECHR

Let us finally consider a version of the HRO according to which non-consensual ALIs violate recipients’ human rights by violating their rights under Article 12 ECHR. Article 12 ECHR holds that ‘[m] en and women of marriageable age have the right to marry and to found a family, according to the national laws governing the exercise of this right’.^120^ The exercise of this right is subject to national law restrictions on its scope, but any limitations imposed by national laws are subject to ECtHR supervision and ‘must not restrict or reduce the right in such a way or to such an extent that the very essence of the right is impaired’.^121^

ALIs may in some recipients reduce their testosterone levels to such a degree that recipients lose their ability to have erections and ejaculate. If offenders for whom ALIs have this effect are required to undergo the intervention for a prolonged time period, ALIs may impede their ability to conceive biological children.^122^

Of course, Article 12 claims are only possible for offenders within a certain (reproductive) age span, who have sex with women, and for whom ALIs have physiological effects of the kind that impedes unassisted reproduction. Some ALI recipients are still able to have an erection and ejaculate and therefore reproduce. In respect of these offenders, then, there is no potential Article 12 breach.^123^ In addition, for some sex offenders, Article 12 may not be a tangible right anyway, because there is no set of circumstances in which they would be able to have a family of this kind, for example, because their offences and circumstances are such that they would (in the context of English law) be subject to care proceedings or Sexual Harm Prevention Orders taking their children away from them if they had them. Nevertheless, there is plausibly a substantial class of offenders who fall within the category whose Article 12 rights may potentially be affected by ALIs.

Some of the plausibility of an Article 12 claim will turn on the length of the intervention and its relation to the reproductive lifespan of the parties affected. In the case of pharmacological ALIs, any effects that prevented reproduction would be reversible once ALIs had been discontinued. Preventing an offender from exercising his right to found a family may be Article 12 compliant if temporary, ‘as long as the length of the treatment does not completely undermine the essence of the right’.^124^ Here, again, proportionality is key. In *Dickson v United Kingdom,*^125^ an imprisoned offender and his wife argued that the refusal of his prison to facilitate the transfer of his sperm to a private fertility clinic at which his wife could undergo *in vitro* fertilisation violated both of their Article 8 and 12 rights. The offender’s wife would be 51 years old at the earliest possible time of his release. The English Court of Appeal accepted that the Dicksons’ need for artificial insemination was outweighed by other factors, including that ‘their relationship had yet to be tested in the normal environment of daily life, making it difficult to assess whether it would continue after Mr Dickson’s release’ (the couple had met while both incarcerated, through an inter-prison pen-pal programme), concerns for the welfare of the future child and public concern that the punitive and deterrent elements of Dickson’s sentence would be undermined by making this service available to him and his wife. This decision was upheld by the Fourth Section of the ECtHR, but struck down upon reference to the Grand Chamber of the ECtHR. The Grand Chamber considered the Dicksons’ Article 8 rights only, since they found it unnecessary to consider Article 12 after having found Article 8 violations. It emphasised that incarceration did not cause individuals to lose their human rights and that any interference with individuals’ Article 8 rights thus must be justified and proportionate. The Court rejected the argument that making artificial insemination available to the Dicksons would offend public opinion and undermine the punitive and deterrent elements of the offender’s sentence but accepted that the state could legitimately ‘take steps to maintain public confidence in the penal system’ and take into account the welfare of the future child.^126^ However, the Court held that the requirement for the applicants to demonstrate that their case was exceptional set too high a threshold for access to artificial insemination and precluded appropriate balancing of the competing interests.^127^ It therefore failed the proportionality inquiry.

Because of Mrs Dickson’s age, a refusal to make artificial insemination available to the couple would not only delay, but altogether eliminate, their chance of founding a family together. *Dickson* can therefore be distinguished from the earlier case of *R v Secretary of State for the Home Department, ex parte Mellor*, in which the English Court of Appeal held a decision not to make facilities for artificial insemination available to be justifiable and proportionate under Article 8(2) and not a breach of Article 12, on grounds that it did not *prevent* the offender and his wife from founding a family but merely *delayed* it. Lord Phillips MR noted that:

Imprisonment is incompatible with the exercise of conjugal rights and consequently involves an interference with the right to respect for family life under Article 8 and with the right to found a family under Article 12. This restriction is ordinarily justifiable under the provisions of Article 8(2).^128^

His Lordship went on to note that ‘[e]xceptional circumstances may require the normal consequences of imprisonment to yield, because the effect of its interference with a particular human right is disproportionate’.^129^ But, he held, ‘it seems to be rational that the normal starting point should be a need to demonstrate that, if facilities for artificial insemination are not provided, the founding of a family may not merely be delayed but prevented altogether’.^130^ Since the offender’s wife in *Mellor* would only be 31 years of age at the time of his release, a delay, the Court held, did not constitute disproportionate interference with his or his partner’s rights.

As long as ALIs are a temporary measure not extending across the offender’s (or his partner’s) entire reproductive lifespan, then, the interference with both parties’ Article 8(1) rights may be accepted as justifiable and proportionate under Article 8(2) and as Article 12-compliant. Moreover, (very) long-term ALI use could be made ECHR-compliant, it seems, by a policy that allowed for artificial insemination facilities to be made available to offenders in exceptional circumstances, per *Mellor.* Indeed, it might be argued that ALI use, even when it prevents an offender from procreating, merely restricts the range of means by which he can found a family, by blocking off (temporarily) one of the avenues for family founding (natural conception). To say that offenders undergoing ALIs are *prevented* from founding a family, then, seems too strong a claim.^131^ Article 12 compliance could be achieved, it seems, by not blocking off other means to founding a family, such as adoption or assisted reproduction, by, for example, giving offenders the option of freezing sperm prior to commencing ALIs.^132^

HRO proponents may object that even if there *exist* other ways in which offenders could found a family, such options may be unavailable to them. Restrictions may prevent individuals convicted of sex offences from adopting, and assisted reproduction is not available to all who wish to use it and may not be available to sex offenders serving a sentence. Both assisted reproduction and adoption (unlike natural conception) are costly, which may preclude offender access. If alternative means of founding a family are also unavailable or severely restricted, long-term ALI use may disproportionately interfere with the offender’s (and his partner’s) Article 12 rights. But it seems that in this respect ALIs are again not necessarily more restrictive than traditional responses to criminal offending, such as incarceration. As we saw in *Dickson* and *Mellor,* incarceration also restricts offenders’ (and their partners’) ability to found a family. ALIs, we might argue, may in fact be less restrictive, depending on how we construe Article 12. If construed with an emphasis on the *conception* of a biological child element of ‘founding a family’, ALIs may be more restrictive than incarceration, because ALI use may require offenders to freeze sperm prior to commencing treatment, while an incarcerated individual could presumably have his sperm retrieved for the purposes of insemination at any time while serving his sentence. If instead construed with an emphasis on the offender’s ability to be part of, or play a role in, *raising* a family, however, ALIs are arguably less restrictive than incarceration. The latter interpretation seems more compelling. As Connie Rosati argues,

Surely the right to control one’s reproduction is not all about the freedom to decide whether to undergo the bare biological processes of conceiving, carrying, and giving birth to babies. Rather, the right concerns most centrally the freedom a person must have to decide whether and when to enter into the intimacy of a parental relationship, with all the rewards, hardships, and obligations that this relationship entails. The loss that a person incurs when her right to control her reproduction has been violated is thus not, in the case in which she wishes to have children, a mere loss of biological function or experience, but a loss of the freedom to nurture and rear her children.^133^

This seems even more obvious when we are concerned with men’s role in founding a family, which would, on the first interpretation, not seem very significant or meaningful. In *Dickson*, the Grand Chamber emphasised the legitimacy of taking into account ‘the impact on the as-yet unconceived child of the prolonged absence from its life of its father, due to his incarceration’.^134^ If ALIs might enable offenders to live outside prison, ALIs may be less restrictive in this respect. Also in respect of Article 12, then, it is far from clear that ALIs are more problematic than traditional methods, from the point of view of offenders’ human rights.

## 5. CONCLUSION

According to the HRO—a common objection to the use of anti-libidinal interventions in sex offenders—(at least) non-consensual ALIs violate recipients’ human rights. In this article I have sought to explicate and evaluate this objection in relation to the ECHR. I have argued that the objection, as it is often stated by its proponents, is unlikely to succeed under the ECHR. The rights that seem most likely to bear on ALI provision—Articles 3, 8 and 12—are unlikely to prohibit many instances of ALI use—also when it is non-consensual. In the face of this argument, the objection’s proponents could either reject the ECHR framework but maintain that ALIs violate human rights or restate their objection so that it better fits this framework. Under the first option, they might develop arguments to the effect that the ECHR should be construed in some other way or that we should rely on some other human rights framework or that some other human right should be recognised in addition to the ones protected by the ECHR.^135^ Another option is to restate the objection such that it better fits the ECHR framework. The preceding analysis has pointed to some ways in which proponents might do so. This would involve diverting focus from offender consent and towards other factors, relating both to the nature of ALIs and the nature of the relevant rights under the ECHR. In respect of Article 3, this involves engaging with whether ALIs are used in ways that seem more correctional (aiming at public protection through the prevention of recidivism) than therapeutic. If this is the case, it may be implausible to construe ALI use as medically necessary, which may give HRO proponents a stronger case under Article 3. In respect of Articles 8 and 12, restating the objection would involve engaging more closely with the balancing of the rights/interests of individuals and state/public interests at the heart of these rights. It is not sufficient to show that offenders possess (retain) the relevant human rights or that ALIs interfere with those rights; in order to successfully object to ALIs, it must also be shown that the interference with offender rights is disproportionate. Given that ALI recipients, as a result of their criminal offending, are already liable to be treated in some ways in which it would not be permissible to them but for their offence, this would seem to generate at least some obligation on critics of ALIs to demonstrate that ALIs are sufficiently more burdensome than traditional responses to offending, such that the former is a disproportionate measure when the latter is not.

## Footnotes

1 The focus in this paper is on ALI use in male sex offenders, but some of my arguments, especially regarding Articles 3 and 8 ECHR, will apply also to other types of biomedical interventions used for crime-prevention purposes and to other types of offenders.

2 Khan et al., ‘Pharmacological interventions for those who have sexually offended or are at risk of offending’ (2015) *Cochrane Database of Systematic Reviews* 2 at 6–7. Examples of hormonal ALIs include medroxyprogesterone acetate (MPA), which is used in the United States, and cyproterone acetate (CPA) and gonadotropin-releasing hormone agonists or analogues (GnRH), more commonly used in European jurisdictions. Examples of non-hormonal ALIs include anti-psychotics and serotonergic antidepressants (SSRIs).

3 Thibaut et al., ‘The World Federation of Societies of Biological Psychiatry (WFSBP) guidelines for the biological treatment of paraphilias’ (2010) 11 *The World Journal of Biological Psychiatry* 604.

4 Scott and Holmberg, ‘Castration of Sex Offenders: Prisoners’ Rights Versus Public Safety’ (2003) 31 *Journal of the American Academy of Psychiatry and the Law* 502. For a discussion of the different types of regimes under which ALIs may be provided, see Forsberg, ‘Crime-preventing Neurointerventions and the Law: Learning from Anti-libidinal Interventions’ in Birks and Douglas (eds), *Treatment for Crime. Philosophical Essays on Neurointerventions in Criminal Justice* (2018) 44.

5 Shaw, ‘Sex offenders in pilot drugs trial’, *BBC News,* 11 June 2012, available at www.bbc.com/news/uk-18402203 [last accessed 1 December 2020]; Darjee, ‘Medical treatment of sexual offenders. Protocol and guidance for the referral, assessment and treatment of sexual offenders managed by criminal justice social work and the prison service in Scotland’, available at www.forensicnetwork.scot.nhs.uk/documents/medication%20for%20sex% 20offenders%20protocol.pdf [last accessed 1 December 2020].

6 Examples of such mental health legislation are the Mental Health Act 1983 in the UK, and the Forensic Mental Care Act 1991: 1129 in Sweden.

7 For example: Cal. Pen. Code §645 (California), Fla. Stat. §794.0235 (1997) (Florida), and La. Rev. Stat. Ann. §15:538(C)(2)(b) (2009) (Louisiana).

8 See Dhingra, ‘PMO asks Women & Child ministry to examine demand for chemical castration for child rapists’, *The Print* (India)*,* 4 May 2018.

9 Douglas, ‘Criminal Rehabilitation Through Medical Intervention: Moral Liability and the Right to Bodily Integrity’ (2014) 18 *The Journal of Ethics* 101. For example, it has been suggested that SSRIs might reduce aggression, see Bond, ‘Antidepressant Treatments and Human Aggression’ (2005) 526 *European Journal of Pharmacology* 218; Nevels et al., ‘Psychopharmacology of Aggression in Children and Adolescents with Primary Neuropsychiatric Disorders: A Review of Current and Potentially Promising Treatment Options’ (2010) 18 *Experimental and Clinical Psychopharmacology* 184 and that Divalproex might reduce impulsiveness, see Donovan et al., ‘Divalproex Treatment for Youth with Explosive Temper and Mood Lability: A Double-Blind, Placebo-Controlled Crossover Design’ (2000) 157 *American Journal of Psychiatry* 818.

10 Rosati, ‘A Study of Internal Punishment’ (1994) *Wisconsin Law Review* 123.

11 Most HRO proponents appear to assume that offender consent makes a difference to whether ALIs violate recipients’ human rights. See Harrison and Rainey, ‘Morality and Legality in the Use of Antiandrogenic Pharmacotherapy with Sexual Offender*s*’ in Boer et al. (eds), *International Perspectives on the Assessment and Treatment of Sexual Offenders: Theory, Practice and Research* (2011) 627; Rainey, ‘Human Rights and Sexual Offenders’ in Harrison and Rainey (eds), *The Wiley-Blackwell Handbook of Legal and Ethical Aspects of Sex Offender Treatment and Management* (2013) 18; Harrison and Rainey, ‘Suppressing Human Rights? A Rights-based Approach to the Use of Pharmacotherapy with Sex Offenders’ (2009) 29 *Legal Studies* 47; Rainey, ‘Dignity and Dangerousness: Sex Offenders and the Community—Human Rights in the Balance?’ in Harrison (ed), *Managing High Risk Sex Offenders in the Community: Risk Management, Treatment and Social Responsibility* (2010) 269; Rainey and Harrison, ‘Pharmacotherapy and Human Rights in Sexual Offenders: Best Friends or Unlikely Bedfellows?’ (2008) 32 *Sexual Offender Treatment* 1. Lando Kirchmair argues that biomedical interventions used for crime-prevention purposes violate the human rights of offenders who receive them without validly consenting, see Kirchmair, ‘Objections to Coercive Neurocorrectives for Criminal Offenders–Why Offenders’ Human Rights Should Fundamentally Come First’ (2019) 38 *Criminal Justice Ethics* 19.

12 See, for example, Griffin, *On Human Rights* (2008); Sen, ‘Human Rights and the Limits of Law’ (2006) 27 *Cardozo Law Review* 2913; Raz, ‘Human Rights Without Foundations’ in Tasioulas and Besson (eds), *The Philosophy of International Law* (2010) 321; Searle, *Making the Social World. The Structure of Human Civilization*, (2010). Note Allen Buchanan’s argument that philosophers have neglected international human rights law in their engagement with the practice of human rights, see Buchanan, *The Heart of Human Rights* (2017). For a response, see Tasioulas, ‘Exiting the Hall of Mirrors: Morality and Law in Human Rights’ in Campbell and Bourne (eds), *Political and Legal Approaches to Human Rights* (2017) 73.

13 Article 5 UDHR holds that ‘[n]o one shall be subjected to torture or to inhuman or degrading treatment or punishment’, and Article 3 ECHR holds that ‘[n]o one shall be subjected to torture or to inhuman or degrading treatment or punishment’. Article 12 UDHR holds that ‘[n]o one shall be subjected to arbitrary interference with his privacy, family, home or correspondence, nor to attacks upon his honour and reputation. Everyone has the right to the protection of the law against such interference or attacks’, and Article 8 ECHR holds that, ‘[e]veryone has the right to respect for his private and family life, his home and his correspondence’, and under Article 8(2) ECHR, ‘t]here shall be no interference by a public authority with the exercise of this right except such as is in accordance with the law and is necessary in a democratic society in the interests of national security, public safety or the economic wellbeing of the country, for the prevention of disorder or crime, for the protection of health or morals, or for the protection of the rights and freedoms of others’. Article 16 UDHR holds that ‘[m]en and women of full age, without any limitation due to race, nationality or religion, have the right to marry and found a family’, and Article 12 ECHR holds that ‘[m]en and women of marriageable age have the right to marry and to found a family, according to the national laws governing the exercise of this right’. Article 9 UDHR holds that ‘[e]veryone has the right to freedom of thought, conscience and religion; this right includes freedom to change his religion or belief, and freedom, either alone or in community with others and in public or private, to manifest his religion or belief in teaching, practice, worship and observance’, and Article 9(1) ECHR holds that ‘[e]veryone has the right to freedom of thought, conscience and religion; this right includes freedom to change his religion or belief and freedom, either alone or in community with others and in public or private, to manifest his religion or belief, in worship, teaching, practice and observance’.

14 Prima facie similar because the substance of the ECHR goes beyond the articles, since the ECtHR is responsible for maintaining the Convention as a ‘living instrument’, see, for example, *Tyrer v United Kingdom* App No 5856/72, Merits, 25 April 1978 at para 31; *Demir and Baykara v Turkey* App No 34503/93, Merits and Just Satisfaction, 12 November 2008 at para 146.

15 It also seems more fruitful to engage with the ECHR rather than other, legally binding international human rights treaties, such as the International Covenant on Civil and Political Rights (ICCPR) on the question of the human rights compliance of non-consensual medical interventions such as ALIs. The ICCPR stipulates some of the same rights as the ECHR. For example, Article 7 ICCPR holds that ‘[n]o one shall be subjected to torture or to cruel, inhuman or degrading treatment or punishment’, including non-consensual ‘medical or scientific experimentation’. Article 7 ICCPR seems relevantly similar to the right not to be subject to torture or inhuman or degrading treatment prescribed by Article 3 ECHR. However, the notional protection of the ICCPR is procedurally and substantively less extensive than the ECHR. Procedurally, not all states parties to the ICCPR have signed and ratified its First Optional Protocol providing an individual complaint mechanism for violations of the ICCPR rights, and several states have acted to limit the jurisdiction of the UN Human Rights Committee (HRC) to hear individual ICCPR complaints. This includes many parties to the ECHR who do not recognise the jurisdiction of the HRC for matters within the scope of the ECHR. This is important for our purposes, because as a consequence, there is a lack of jurisprudence of the HRC on the human rights objection to ALIs or jurisprudence from which useful analogies may be drawn. Substantively, we will see that more grounds exist on which to advance the human rights objection to ALIs under the ECHR. Where there is overlap between the ICCPR and the ECHR rights, I would suggest that the arguments advanced in respect of the latter are applicable *mutatis mutandis* to the former.

16 Some of the most developed accounts of the HRO are found in Harrison and Rainey, ‘Morality and Legality in the Use of Antiandrogenic Pharmacotherapy with Sexual Offenders’, supra n 11; Rainey, ‘Human Rights and Sexual Offenders’, supra n 11; Harrison and Rainey, ‘Suppressing Human Rights?’, supra n 11.

17 See Rosati, supra n 10 at 124.

18 Harrison and Rainey, ‘Morality and Legality in the Use of Antiandrogenic Pharmacotherapy with Sexual Offenders’, supra n 11; Rainey, ‘Human Rights and Sexual Offenders’, supra n 11; Harrison and Rainey, ‘Suppressing Human Rights?’, supra n 11; Rainey, ‘Dignity and Dangerousness’, supra n 11; Rainey and Harrison, ‘Pharmacotherapy and Human Rights in Sexual Offenders’, supra n 11; Akbaba, *The Permissibility of Pharmacotherapy for Paedophilic Sex Offenders in Light of the Rights Protected under the European Convention on Human Rights* (PhD thesis, University of Leicester, 2015).

19 See, for instance, Fennell, *Treatment Without Consent: Law, Psychiatry and the Treatment of Mentally Disordered People Since 1845* (1996); McTernan, ‘Those Who Forget the Past: An Ethical Challenge from the History of Treating Deviance’, in Birks and Douglas (eds), *Treatment for Crime. Philosophical Essays on Neurointerventions in Criminal Justice* (2018) 274.

20 Examples of the HRO expressed by academic commentators include: Harrison and Rainey, ‘Morality and Legality in the Use of Antiandrogenic Pharmacotherapy with Sexual Offenders’, supra n 11; Rainey, ‘Human Rights and Sexual Offenders’, supra n 11; Harrison and Rainey, ‘Suppressing Human Rights?’, supra n 11; Rainey, ‘Dignity and Dangerousness’, supra n 11; Rainey and Harrison, ‘Pharmacotherapy and Human Rights in Sexual Offenders’, supra n 11; Kirchmair has made a similar argument according to which biomedical interventions, which could include ALIs, used for crime-prevention purposes violate the human rights of offenders who receive them, see Kirchmair, ‘Objections to Coercive Neurocorrectives for Criminal Offenders’, supra n 11.

21 Amnesty International, ‘Indonesia: Halt Chemical Castration’, 13 October 2016, available at www.amnesty.org/en/latest/news/2016/10/indonesia-halt-chemical-castration/ [last accessed 20 January 2021]; Madison Park, ‘Using chemical castration to punish child sex crimes’, CNN, 5 September 2012, available at https://edition.cnn.com/2012/09/05/health/chemical-castration-science/index.html [last accessed 20 January 2021]; Amnesty International, ‘Pakistan: Cruel and inhuman chemical castration punishment will not fix flawed system’, 16 December 2020, available at https://www.amnesty.org/en/latest/news/2020/12/pakistan-cruel-and-inhuman-chemical-castration-punishment-will-not-fix-flawed-system/ [last accessed 20 January 2021].

22 For some renditions of the HRO in popular press: *The Guardian* cites a source who expresses the HRO: ‘We oppose all corporal punishment, because it violates the bodily integrity and the human rights of a person’ in Letsch, ‘Chemical Castration of Sex Offenders in Turkey Condemned by Women’s Groups’, *The Guardian*, 15 August 2016, available at www.theguardian.com/global-development/2016/aug/15/turkey-chemical-castration-law-sex-offenders-condemned-womens-groups [last accessed 1 December 2020]; *The Jakarta Post* states describes how ‘Institute for Criminal Justice Reform researcher Anggara demanded that the government and the House of Representatives drop the plan, the implementation of which, she said, would constitute a clear violation of human rights’ in Sundaryani, ‘Chemical castration “would violate human rights of sex offenders”’, *The Jakarta Post*, 31 October 2015, available at www.thejakartapost.com/news/2015/10/31/chemical-castration-would-violate-human-rights-sex-offenders.html [last accessed 1 December 2020].

23 Amnesty International, ‘Indonesia: Halt Chemical Castration’, supra n 21.

24 Amnesty International, ‘Moldova: Forced Chemical Castration Constitutes Inhuman Treatment’, supra n 21.

25 Council of Europe, European Committee for the Prevention of Torture and Inhuman or Degrading Treatment, *Report to the Czech Government on the Visit to the Czech Republic of the European Committee for the Prevention of Torture and Inhuman or Degrading Treatment from 27 March–7 April, 21–24 June 2006*, CPT/Inf (2007) 32, 12 July 2007 at para 109.

26 Rainey, ‘Human Rights and Sexual Offenders’, supra n 11 at 33. See also Rainey and Harrison, ‘Pharmacotherapy and Human Rights in Sexual Offenders’, supra n 11.

27 Harrison and Rainey, ‘Morality and Legality in the Use of Antiandrogenic Pharmacotherapy with Sexual Offenders’, supra n 11 at 640.

28 See, for instance, *Jehovah’s Witnesses of Moscow and others v Russia App* No 302/02, Merits and Just Satisfaction, 10 June 2010, at para 135.

29 Outside the mental health context, health professionals must gain valid consent for medical treatment to be lawful (*Aintree University Hospitals NHS Foundation Trust v James* [2013] UKSC 67, [2014] AC 591; *Airedale NHS Trust v Bland* [1993] 1 AC 789 (HL)). The conditions for valid consent are adequate information (*Chatterton v Gerson* [1981] QB 432), decision-making capacity (Mental Capacity Act 2005, ss 1–3) and the absence of undue influence (*Re T (Adult: Refusal of Treatment)* [1993] Fam 95 (CA Civ)). In respect of individuals who lack decision-making capacity, consent is deemed by operation of law, when the interventions are in the patient’s best interests (MCA 2005, ss 4–5).

30 Thus, objections to ALIs centre on offering ALIs as an alternative to incarceration being coercive, rendering consent invalid, see Vanderzyl, ‘Castration as an Alternative to Incarceration: An Impotent Approach to the Punishment of Sex Offenders’ (1994) 15 *Northern Illinois University Law Review* 107; Green, ‘Depo-Provera, Castration, and the Probation of Rape Offenders (1986) 12 *University of Dayton Law Review* 1, whereas defences respond that, although offenders face a strong incentive to consent, this does not invalidate consent, see Rosati, supra n 10. For some exceptions, see Ryberg, ‘Is Coercive Treatment of Offenders Morally Acceptable? On the Deficiency of the Debate’ (2015) 9 *Criminal Law and Philosophy* 619; Ryberg, ‘Punishment, Pharmacological Treatment, and Early Release’ (2012) 26 *International Journal of Applied Philosophy* 231; Douglas, ‘Criminal Rehabilitation Through Medical Intervention’, supra n 9.

31 Harrison and Rainey, ‘Morality and Legality in the Use of Antiandrogenic Pharmacotherapy with Sexual Offenders’, supra n 11 at 640. The Council of Europe’s Committee for the Prevention of Torture made the more modest claim that ‘consent is hardly reconcilable with a situation in which the options open to an individual are extremely limited: surgical castration or possible indefinite confinement in a psychiatric hospital’, Council of Europe, *Report to the Czech Government*, supra n 25 at para 109. The assumption that ALIs offered as a condition of parole or alternative to incarceration amounts to coerced (and therefore invalid) consent is controversial. For discussions regarding surgical castration and the effects of ‘coercive offers’ on consent, see McMillan, ‘The Kindest Cut? Surgical Castration, Sex offenders and Coercive Offers (2014) 40 *Journal of Medical Ethics* 583, and regarding coercive offers and neurointerventions, see Pugh, ‘Coercion and the Neurocorrective Offer’ in Birks and Douglas (eds), *Treatment for Crime* (2018) 94; Feinberg, *The Moral Limits of the Criminal Law Volume 3: Harm to Self* (1989) at Chapter 3. For a liability justification for treating offenders’ consent as valid, see McMahan, ‘Moral Liability to “Crime-Preventing Neurointervention”’ in Birks and Douglas (eds), *Treatment for Crime. Philosophical Essays on Neurointerventions in Criminal Justice* (2018) 117. The law does not support the claim that offering ALIs as a condition of parole amounts to coercion vitiating consent *tout court*; being incarcerated/detained does not per se prevent prisoners from giving effective consent, see *Freeman v Home Office* [1984] 1 All ER 524 CA at 556; *R (H) v Mental Health Review Tribunal* [2007] EWHC 884); *Janiga v Usti Nad Labem Regional Court, Czech Republic* [2011] EWHC 553 (Admin), [2011] All ER (D) 236.

32 When the State or agents of the State impose the proscribed treatment.

33 *Chahal v United Kingdom* Application No 22414/93, Merits and Just Satisfaction, 15 November 1996, at para 79.

34 *R v Secretary of State for the Home Department, ex parte Limbuela* [2005] UKHL 66, [2005] 3 WLR 1014 at 77. See also *Gäfgen v Germany* Application No 22987/05, Merits and Just Satisfaction, 1 June 2010. For discussions regarding Article 3 and the idea of absoluteness, see, for instance, Graham, ‘*Jeanty v Belgium*: Saving Lives Provides (another) Exception to Article 3 ECHR’ (2021) 21(1) *Human Rights Law Review*; Greer, ‘Is the Prohibition against Torture, Cruel, Inhuman and Degrading Treatment Really “Absolute” in International Human Rights Law? A Reply to Graffin and Mavronicola’ (2018) 18(2) *Human Rights Law Review* 297; Mavronicola, ‘Is the Prohibition Against Torture and Cruel, Inhuman and Degrading Treatment Absolute in International Human Rights Law? A Reply to Steven Greer’ (2017) 17 *Human Rights Law Review* 479; Greer, ‘Is the Prohibition against Torture, Cruel, Inhuman and Degrading Treatment Really “Absolute” in International Human Rights Law?’ (2015) 15 *Human Rights Law Review* 101; Smet, ‘Conflicts between Absolute Rights: A Reply to Steven Greer’ (2013) 13 *Human Rights Law Review* 469; Mavronicola, ‘What is an “absolute right”? Deciphering Absoluteness in the Context of Article 3 of the European Convention on Human Rights’ (2012) 12 *Human Rights Law Review* 723; Palmer, ‘A Wrong Turning: Article 3 ECHR and Proportionality’ (2006) 65 *Cambridge Law Journal* 438, 446; Möller, ‘Balancing and the Structure of Constitutional Rights’ (2007) 5 *International Journal of Constitutional* Law 453, 466.

35 *Chahal*, supra n 33*; Limbuela,* supra n 34.

36 *Ireland v United Kingdom* Application No 5310/71, Merits and Just Satisfaction,18 January 1978, at para 162.

37 *R (on the application of B) v Dr SS, Responsible Medical Officer, and Others* [2005] EWHC 1936 (Admin), [2005] EWHC 1936.

38 *Ireland v UK*, supra n 36.

39 Khan et al., supra n 2 at 24–5.

40 Harrison and Rainey, ‘Suppressing Human Rights?’, supra n 11, at 65. Non-consensual surgical castration has also been found to be cruel and unusual punishment by the US Supreme Court (*State v. Brown*, 326 S.E.2d 410 (S.C. 1985)), but consensual surgical castration may be offered as a voluntary alternative to ALIs in, for example, Texas, see Texas Government Code Ann § 508.226. (2001).

41 ALIs can be administered as surgical implants, but this is less common. Mental Health Act 1983, Section 57 restricts use of hormonal ALI surgical implants.

42 *Jalloh v Germany* Application No 54810/00, Merits and Just Satisfaction,11 July 2006, at para 76.

43 Ibid. at para 68–69. See also *Dvořáček v the Czech Republic* Application No 1297/13, Merits, 6 November 2014, at para 86.

44 *Bouyid v Belgium* Application No 23380/90, Merits and Just Satisfaction, 29 September 2015, at paras 100–102.

45 *Jalloh*, supra n 42.

46 *Bouyid*, supra n 44 at para 86.

47 A Cochrane systematic review found that in the small sample of ALI trials that included information regarding adverse events (n = 6), the most significant negative side-effect (of MPA and CPA) was weight gain—participants gained an average of 3.1 kg during trials, see Khan et al., supra n 2 at 19. Two other side-effects (of MPA) were statistically significant in one of the studies included: depression and excess salivation, see Khan et al., supra n 2 at 22. The most severe side-effects (extra-pyramidal movement disorders and drowsiness) were reported in a trial of anti-psychotic medication (n = 12)’, see Khan et al., supra n 2 at 2. In one small study some participants who used two types of anti-psychotic medication reported blurred vision, photosensitivity, dry mouth and impaired exercise tolerance, see Khan et al., supra n 2. In another study, undescribed side-effects led some participants to discontinue intramuscular MPA after three to five injections. Lewis et al.’s systematic review of all published studies until 2015 on GnRH agonists in male sex offenders observe that side-effects most commonly reported were ‘hot flushes, reduction in testicular volume, weight gain, injection site pain and gynaecomastia, painful erections, reduced body hair and depression’, although they note that ‘the exact number of subjects reporting the effects was not always present’, see Lewis et al., ‘Gonadotrophin-releasing Hormone Agonist Treatment for Sexual Offenders: a Systematic Review’ (2017) 31 *Journal of Psychopharmacology* 1281 at 1287). Koo et al., ‘Treatment Outcomes of Chemical Castration on Korean Sex Offenders’ (2013) 20 *Journal of Forensic Legal Medicine* 563 provide an indication of how often side-effects occur in GnRH agonists (n = 56): hot flushes: 45 per cent, weight gain: 29 per cent, testis size reduction: 24 per cent, depressive mood: 21 per cent, injection site pain: 18 per cent, myalgia: 11 per cent.

48 Fennell, ‘Sex Offenders, Consent to Treatment and the Politics of Risk’ in Harrison and Rainey (eds), *The Wiley-Blackwell Handbook of Legal and Ethical Aspects of Sex Offender Treatment and Management* 38 at 50.

49 Harrison and Rainey, ‘Suppressing Human Rights?’, supra n 11 at 65.

50 Lippi and van Staden compile a list of all possible effects of CPA on sex offenders, which include, among others, effects that may affect personality: fatigue, depressive symptoms or disorders, emotional disturbances, see Lippi and van Staden, ‘The use of cyproterone acetate in a forensic psychiatric cohort of male sex offenders and its associations with sexual activity and sexual functioning’ (2017) 23 *South African Journal of Psychiatry* 982. The Cochrane review also includes a review of how often psychological/psychiatric side-effects occur, like anxiety, feelings anger or aggression or suicide attempts. It seems that, depending on the drug used, anger and aggression are reduced, see Khan et al., supra n 2 at 19–20, with patients being more calm and less irritable. No deaths or suicide attempts were reported in any study, see Khan et al., supra n 2 at 2.

51 *Grare v France* Application No 18835/91, Commission Decision, 2 December 1992.

52 Rainey, ‘Human Rights and Sexual Offenders’, supra n 11 at 33.

53 Rainey and Harrison, ‘Pharmacotherapy and Human Rights in Sexual Offenders’, supra n 11 at 3.

54 *Herczegfalvy v Austria* Application No 10533/83, Merits and Just Satisfaction, 24 September 1992, at para 83.

55 Ibid. at para 82.

56 Harrison and Rainey, ‘Suppressing Human Rights?’, supra n 11 at 73. Legal regimes in some other jurisdictions, such as the Cal. Pen. Code §645, allow for blanket provision of ALIs of this kind.

57 The likelihood here might depend on how exactly they take the diagnoses into account and how much.

58 Fennell, ‘Sex Offenders, Consent to Treatment and the Politics of Risk’, supra n 48 at 51.

59 In *R v Mental Health Act Commission, ex parte W* (1988) 9 BMLR 77 (CA) at 84–85, it was held that the sexual offending behaviour was ‘inextricably linked with the mental disorder’ and that interventions targeting the sexual offending behaviour, therefore, were ‘treatment for mental disorder’.

60 Mental Health Act 1983, Section 3(2)(b).

61 *Dvořáček*, supra n 43 at 94. The reference to the applicant’s conduct as potentially justifying the measures taken here may sound like a public protection justification, which would sit uneasily with the unqualified nature of Article 3. However, it may be that the reference to the applicant’s conduct refers to what is also said in *Jalloh* regarding the *context* in which the alleged breach of Article 3 occurs being crucial to determining Article 3 breach (*Jalloh,* supra n 42 at paras 70, 76). As noted previously, one of the features to take into account here is the *purpose* of the measure (for example, obtain evidence; medical treatment). This may indicate, then, that the ECtHR agrees with domestic courts that it was necessary for the applicant, taking into account his conduct, to be confined to a hospital in order for the medical intervention to have the desired effects. It may also be that the applicant’s conduct, while perhaps not a truthmaker for mental disorder, may be epistemically relevant, by providing evidence for it. In *Bure*š *v the Czech Republic* Application No 37679/08, Merits and Justification, 18 October 2012, the European Court discussed the general tests for the administration of restraint treatment for a mentally ill applicant, at paras 91–104.

62 Khan et al., supra n 2.

63 *R (on the application of Wilkinson) v Responsible Medical Officer Broadmoor Hospital* [2001] EWCA Civ 1545, [2002] 1 WLR 419 at para 30.

64 In *R (on the application of B) v Haddock (Responsible Medical Officer)* [2006] EWCA Civ 961, [2006] All ER (D) 137 (Jul).

65 *R (N) v M and Others* [2002] EWCA Civ 1789, [2003] 1 WLR 562 at paras 27, 29.

66 For a discussion, see Bartlett, ‘“The Necessity Must be Convincingly Shown to Exist”: Standards for Compulsory Treatment for Mental Disorder under the Mental Health Act 1983’ (2011) 19 *Medical Law Review* 514 at 524–6, 535.

67 ECtHR case law considering positive obligations arising from Article 3 (in relation especially to the state’s obligation to provide (medical) treatment that is not contrary to the prohibition against torture and inhuman/degrading treatment) and dealing with the ‘adequacy’ of medical treatment provided to prisoners may also shed light on how ECtHR evaluates medical interventions and medical expertise, see, for example, *Antonov v Ukraine* Application No 40512/13, Merits and Justification, 22 October 2015; *Wenner v Germany* Application No 62303/13, Merits and Justification, 1 September 2016.

68 See supra n 20. I take correctional to mean interventions used for anti-recidivism, rehabilitation, public protection and such purposes. For a discussion, see Forsberg and Douglas, ‘Anti-Libidinal Interventions in Sex Offenders: Medical or Correctional?’ (2016) 24 *Medical Law Review* 453.

69 Forsberg and Douglas, supra n 68 at 472.

70 The claim here is that it seems to make a consent requirement *less* plausible. A consent requirement may not be completely implausible, for instance, if ALIs are administered for correctional purposes but only on the condition that they also treat a mental disorder in the offender.

71 *Dvořáček*, supra n 43 at para 92 (emphasis added).

72 *RS v Hungary* Application No 65290/14, Merits and Just Satisfaction, 2 July 2019, at paras 65, 69, 72. *Herczegfalvy,* supra n 54 at para 82*; Jalloh*, supra n 42 at paras 76, 82; *Valasinas v Lithuania* Application No 44558/98, Merits and Just Satisfaction, 24 July 2001, at para 117; *Bouyid*, supra n 44 at paras 100–102 *Dvořáček*, supra n 43 at para 92, 94.

73 In *Jalloh*, supra n 42 at para 82 the fact that the medical procedure and emetics were administered for the purposes of obtaining evidence and not for therapeutic reasons was held to be relevant, in *RS v Hungary*, supra n 72, the fact that the catheterisation was not for therapeutic reasons was also considered relevant (para 72), and in *Dvořáček:* the fact that ALIs were administered for medical reasons was considered relevant (*Dvořáček*, supra n 43 at 92, 94).

74 We do not typically impose a consent requirement in respect of correctional interventions, see Forsberg and Douglas, supra n 68 at 457.

75 Article 8 ECHR.

76 *Pretty v United Kingdom* Application No 2346/02, Merits, 29 April 2002.

77 Herring and Wall, ‘The Nature and Significance of the Right to Bodily Integrity’ (2017) 76 *The Cambridge Law Journal* 566 at 574. Although ECtHR judgments’ interpretations (and those of domestic courts) of body integrity and autonomy and their relation are often conceptually unclear. One example of this noted by Herring and Wall is how in *Price v United Kingdom*, ‘there was no touching or direct interference with the body of the individual, yet this was regarded as an interference in bodily integrity’, 575. See *Price v United Kingdom* Application No 33394/96, Merits and Justification, 10 July 2001, 169.

78 *Pretty*, supra n 76 at para 61.

79 Ibid.

80 *X v Austria* Application No 8278/78, Commission Decision, 13 December 1979, at para 3.

81 *YF v Turkey* Application No 24209/94, Merits and Just Satisfaction, 22 July 2003, at para 43.

82 *Pretty,* supra n 76; *Haas v Switzerland* Application No 31322/07, Merits and Just Satisfaction, 20 January 2011. For a discussion, see Black, ‘Refusing Life-Prolonging Medical Treatment and the ECHR’ (2018) 38 *Oxford Journal of Legal Studies* 299.

83 Douglas et al., ‘Coercion, Incarceration and Chemical Castration: An Argument from Autonomy’ (2013) 10 *Journal of Bioethical Inquiry* 395; Ryberg, ‘Punishment, Pharmacological Treatment, and Early Release’, supra n 30. Rosati argues that internal punishment can be autonomy promoting, see Rosati, supra n 10 at 146. For a similar argument employing Beyleveld and Brownsword’s notion of ‘dignity as empowerment’, see Akbaba, supra n 18 at 99–111, Beyleveld and Brownsword, *Human Dignity in Bioethics and Biolaw* (2001).

84 Douglas notes that it is generally accepted that ‘[a]n important feature of [the moral] right [to bodily integrity] is that it protects against the relevant kinds of bodily interference regardless of what consequences that interference might contingently have, and regardless of what motives might contingently have motivated it’, Douglas, ‘Criminal Rehabilitation Through Medical Intervention’, supra n 9 at 106. I will understand the right to bodily integrity as a right to determine that interferences with one’s bodily integrity to permit.

85 Note for example the line of cases in which English courts have stressed that, even if the patient’s decision is ‘prejudicial’ to their health and well-being, necessary to save their life, or would seemingly enhance their autonomy, their right to refuse treatment, even for idiosyncratic reasons, must be respected: See, for instance, *King’s College Hospital NHS Foundation Trust v C and V* [2015] EWCOP 59.

86 *R (N)*, supra n 65.

87 Fennell, ‘Sex Offenders, Consent to Treatment and the Politics of Risk’, supra n 48 at 51.

88 *R v Mental Health Act Commission, ex parte W*, supra n 59.

89 *Pretty,* supra n 76.

90 *X v Austria*, supra n 80.

91 *Peters v Netherlands* Application No 21132/93, Commission Decision, 6 April 1994.

92 *Acmanne v Belgium* Application No 10435/83, Commission Decision, 10 December 1984.

93 *Grare*, supra n 51.

94 Khan et al., supra n 2 at 1.

95 Gerards, ‘How to Improve the Necessity Test of the European Court of Human Rights’ (2013) 11 *International Journal of Constitutional Law* 466.

96 For instance, *Soering v United Kingdom Application* No 14038/88, Merits and Just Satisfaction, 7 July 1989; *Sporrong and Lönnroth v Sweden Application* No 7151/75, Merits, 23 September 1982.

97 The proportionality test has been applied by the ECtHR in cases such as *Hatton v United Kingdom* Application No 36022/97, Merits and Just Satisfaction, 8 July 2003, at paras 121–122; *Smith and Grady v United Kingdom* Application No 33985/96, Merits, 27 September 1999, at paras 74, 97; *Pretty*, supra n 76 at paras 68, 70, 75–76. For a discussion of proportionality from a judicial perspective, including its history and use in different jurisdictions, see Barak, *Proportionality: Constitutional Rights and Their Limitations* (2012), especially at 175–210.

98 *R (Aguilar Quila and another) v Secretary of State for the Home Department* [2011] UKSC 45, [2012] 1 AC 621.

99 Courts claim proportionality analysis is heuristic/epistemic, but Letsas argues that it is constitutive and involves moral reasoning when thinking about what rights people have (in respect of limb four in particular). See Letsas, ‘Proportionality as Fittingness: The Moral Dimension of Proportionality’ (2018) 71 *Current Legal Problems* 53.

100 *Bank Mellat* [2013] UKSC 39, [2014] AC 700 at para 74.

101 For a discussion over what can be viewed as a necessity, see *Handyside v United Kingdom* Application No 5493/72, Merits, 7 December 1976, at paras 46–49.

102 For a moral defence of the liability justification, see McMahan, ‘Moral Liability to “Crime-Preventing Neurointervention”’, supra n 31.

103 Douglas, ‘Criminal Rehabilitation Through Medical Intervention’, supra n 9 at 107–108.

104 As Douglas notes, ‘[i]t is widely thought that the state may permissibly do things to criminal offenders without their consent that it could not permissibly do to others without (and in some cases even with) consent’, ibid. at 105–6.

105 For discussions of these types of views, see Wellman, ‘The Rights Forfeiture Theory of Punishment’ (2012) 122 *Ethics* 371; Wellman ‘Rights and State Punishment’ (2009) 106 *Journal of Philosophy* 419 and Ross, *The Right and the Good* (1930) at 60–1. For an application to biomedical interventions in crime-prevention, see McMahan, ‘Moral Liability to “Crime-Preventing Neurointervention”’, supra n 31.

106 Douglas, ‘Criminal Rehabilitation Through Medical Intervention’, supra n 9 at 107–8. Incarceration interferes with the right to liberty, explicitly protected by Article 5 ECHR, which provides that ‘Everyone has the right to liberty and security of person. No one shall be deprived of his liberty save in the following cases and in accordance with a procedure prescribed by law’, whereas personal autonomy/bodily integrity is protected by Article 8 ECHR. The protection each right offers is different: Article 8 is a qualified right and any interference with it must survive the proportionality inquiry, whereas Article 5 is a right with exceptions, which means that any interference with it that does not fall within one of the exceptions entails a violation of it. Exceptions include ‘the lawful detention of a person after conviction by a competent court’. Another specified exempted circumstance potentially relevant is ‘(e) the lawful detention of persons for the prevention of the spreading of infectious diseases, of persons of unsound mind, alcoholics or drug addicts or vagrants’.

107 Douglas, ‘Criminal Rehabilitation Through Medical Intervention’, supra n 9 at 111.

108 *Chahal,* supra n 33*; Limbuela,* supra n 34.

109 Douglas, ‘Criminal Rehabilitation Through Medical Intervention’, supra n 9 at 109.

110 Harrison and Rainey, ‘Suppressing Human Rights?’, supra n 11 at 65.

111 Douglas, ‘Criminal Rehabilitation Through Medical Intervention’, supra n 9 at 114; McMahan, ‘Moral Liability to “Crime-Preventing Neurointervention”’, supra n 31. For a discussion of incarceration’s harmfulness and the ECHR, see Ligthart et al., ‘Prison and the Brain: Neuropsychological Research in the Light of the European Convention on Human Rights’ (2019) 10 *New Journal of European Criminal Law* 287. See also Brownlee, ‘A Human Right against Social Deprivation’ (2013) 61 *The Philosophical Quarterly* 199.

112 Douglas, ‘Criminal Rehabilitation Through Medical Intervention’, supra n 9 at 114–5. See also Eyal, ‘Is the Body Special? Review of Cécile Fabre, Whose Body is it Anyway? Justice and the Integrity of the Person’ (2009) 21 *Utilitas* 233.

113 Douglas, ‘Criminal Rehabilitation Through Medical Intervention’, supra n 9 at 115–8. Our environment, Douglas notes, exerts a direct influence on us, including on our minds, and incarcerated individuals frequently suffer mind-altering effects, most clearly seen in solitary confinement, which can cause a number of agency-impeding mental conditions, including things like psychosis, depression, anxiety, anger and poor impulse control, cognitive disturbances, inability to tolerate external stimuli, panic attacks, paranoia, hallucinations and other perceptual disturbances, self-injurious behaviour and so forth. Douglas, ‘Criminal Rehabilitation Through Medical Intervention’, supra n 9 at 117. See, for instance, Haney, ‘Mental Health Issues in Long-term Solitary and “Supermax” confinement’ (2003) 49 *Delinquency* 124; Grassian, ‘Psychiatric Effects of Solitary Confinement’ (2006) 22 *Washington University Journal of Law and Policy* 325; Guenther, *Solitary Confinement. Social Death and its Afterlives* (2013). For a discussion see Ligthart et al., supra n 111.

114 Rosati, supra n 10 at 125.

115 We might worry about cumulative effects, that is, that ALIs, given their (suppose) burdensome features, would constitute double punishment of recipients, if administered to individuals who were already incarcerated. But this is only a worry if ALIs (with these burdensome effects) are administered in addition to prison sentences. ALIs administered in lieu of incarceration would not give rise to any such concerns.

116 Khan et al. found only small trials, most of which were two decades old, and none of which investigated the efficacy of newer drugs. They concluded that ‘[a] lthough there were some encouraging findings’ in some of the studies reviewed, ‘their limitations do not allow firm conclusions to be drawn regarding pharmacological intervention as an effective intervention for reducing sexual offending (Khan et al., supra n 2 at 2).

117 And even then, states might have the authority to use methods that are still under development. See Akbaba, supra n 18 at 239.

118 Pugh and Douglas note the limited effectiveness of incarceration in preventing recidivism, see Pugh and Douglas, ‘Justifications for Non-Consensual Medical Intervention: From Infectious Disease Control to Criminal Rehabilitation’ (2016) 25 *Criminal Justice Ethics* 205 at 220. Mews, Di Bella and Purver, *Impact evaluation of the prison-based Core Sex Offender Treatment Programme*, Ministry of Justice, UK Government (2017), which observed (statistically significant) higher recidivism rates in the follow-up period (on average 8.2 years) among sex offenders who had participated in the cognitive-behavioural psychological intervention programme, compared with the non-participating sex offenders in the control group. Another reply open to HRO proponents, of course, is to argue that she is not committed to defending current incarceration practices or indeed incarceration at all. In analysing the relative burdensomeness of ALIs and traditional correctional practices such as incarceration, we might of course come to the view that neither seems justified or proportionate.

119 Rosati argues that interventions that interfere with bodily integrity could in many cases ‘perfectly well be part of a sentence which, taken as a whole, is proportionate to the severity of the crime committed’, Rosati, supra n 10 at 150. Note that Rosati also argues that ‘internal punishment’ such as ALIs may in fact be *too lenient* to be proportionate from a retributivist point of view (ibid. at 150–2). This would of course not be problematic from the point of view of human rights, but it does indicate that it is far from obvious that interventions that interfere with bodily integrity would always be disproportionately harsh.

120 Article 12 ECHR.

121 *Rees v United Kingdom* Application No 9532/81, Merits, 17 October 1986, at para 50.

122 Harrison and Rainey, ‘Morality and Legality in the Use of Antiandrogenic Pharmacotherapy with Sexual Offenders’, supra n 11 at 646.

123 Harrison and Rainey, ‘Suppressing Human Rights?’, supra n 11 at 71.

124 Rainey and Harrison, ‘Pharmacotherapy and Human Rights in Sexual Offenders’, supra n 11 at 8.

125 *Dickson v United Kingdom* Application No 44362/04, Merits and Just Satisfaction, 4 December 2007.

126 Ibid. at paras 75, 76.

127 Ibid. at para 82.

128 *R (on the application of Mellor) v Secretary of State for the Home Department* [2001] EWCA Civ 472, [2002] QB 13 at 27.

129 Ibid. at 28.

130 Ibid. at 34.

131 Note *Evans v United Kingdom* Application No 6339/05, Merits 10 April 2007, at para 90, in which it was held that the fact that allowing the destruction of embryos created with the applicant’s ex partner’s sperm removed her last remaining possibility of conceiving a genetically related child was insufficient for Article 8 to have been violated, noting the margin of appreciation afforded to member states in respect of such issues.

132 Harrison and Rainey, ‘Morality and Legality in the Use of Antiandrogenic Pharmacotherapy with Sexual Offenders’, supra n 11 at 646.

133 Rosati, supra n 10 at 142–3.

134 *Dickson*, supra n 125 at para 76.

135 For instance, Bublitz and Merkel, ‘Crimes Against Minds: On Mental Manipulations, Harms and a Human Right to Mental Self-Determination’ (2014) 8 *Criminal Law and Philosophy* 51. The idea that ALIs may interfere with recipients’ freedom of thought has also been expressed in the context of ‘ALI statutes’ in the US, see, for example, Stelzer, ‘Chemical Castration and the Right to Generate Ideas: Does the First Amendment Protect the Fantasies of Convicted Pedophiles’ (1997) 81 *Minnesota Law Review* 1339.

